# Early referral and control of disease’s flares prevent Orthopedic and Hand Surgery Indication (OHSI) in a dynamic cohort of Hispanic early rheumatoid arthritis patients

**DOI:** 10.1186/s12891-018-2299-9

**Published:** 2018-10-20

**Authors:** Irazú Contreras-Yáñez, G. Guaracha-Basáñez, E. Díaz-Borjón, M. Iglesias, V. Pascual-Ramos

**Affiliations:** 1 Department of Immunology and Rheumatology, Instituto Nacional de Ciencias Médicas y Nutrición Salvador Zubirán, Vasco de Quiroga 15, Colonia Sección XVI, Belisario Domínguez, 14500 Ciudad de México, CP Mexico; 2 Department of Surgery, Orthopedic Unit, Instituto Nacional de Ciencias Médicas y Nutrición Salvador Zubirán, Mexico City, Mexico; 3 Department of Surgery, Plastic Surgery Unit, Instituto Nacional de Ciencias Médicas y Nutrición Salvador Zubirán, Mexico City, Mexico

**Keywords:** Rheumatoid arthritis, Orthopedic surgery predictors, Disease flares, Early referral

## Abstract

**Background:**

Reconstructive joint surgery is an indicator of poor prognosis in rheumatoid arthritis (RA). Objectives of this study were to describe the incidence rate of orthopedic and hand surgery indication (OHSI) in an ongoing cohort of Hispanic early RA patients treated according to a T2T strategy and to investigate predictors.

**Methods:**

Through February 2018, the cohort comprised 185 patients recruited from 2004 onwards, with variable follow-up, and rheumatic assessments at fixed intervals that included prospective determination of OHSI. Charts were reviewed by a single data abstractor. OHSI incidence rate was calculated. A case-control study nested within a cohort investigated the predictors; cases (OHSI patients) were paired with controls (1:4) according to age, sex and autoantibodies. A logistic regression model included baseline and cumulative (up to OHSI or equivalent) variables related to disease activity, treatment and to persistence with therapy. The IRB approved the study.

**Results:**

Patients from the cohort were predominantly middle-aged (mean ± SD age: 38.5 ± 12.9 years) females (87.6%) with 5.4 ± 2.6 months of disease duration. The cohort contributed to 1538 patient-years of follow-up. Twelve patients received incidental OHSI at a follow-up of 85 ± 44.5 months. The OHSI incident global rate was 8/1000 patient-years. Longer symptom duration at cohort referral (OR: 1.313, 95%CI: 1.02–1.68, *p* = 0.032) and a higher number of flares/patient (OR: 1.608, 95%CI: 1.05–1.61, *p* = 0.015) predicted OHSI. OHSI patients had more severe flares than their counterparts, and the opposite figure was true for mild flares.

**Conclusion:**

Early referral for appropriate management and flare control may prevent OHSI in Hispanic recent-onset RA patients.

## Background

Rheumatoid arthritis (RA) patients from Latin-America present distinctive epidemiological, serological and phenotypic characteristics when compared to Caucasians patients, and these are known to impact patient outcomes [1–3]. In addition, treating RA to target (T2T) has become an internationally agreed standard of good practice [4] although the implementation of such strategy may be restricted in developing countries and non-universal health care systems where adherence to treatment may be dramatically compromised [5, 6].

Despite early and more aggressive treatment guidelines adopted in the last decades, some patients present progressive joint destruction and eventually require a surgical solution. Joint surgery is generally considered an indicator of medical therapy failure and of poor prognosis. In addition, the appropriate orchestration and selection of joint surgical interventions are controversial and problematic for the rheumatologist [7, 8]. Recognition of predictive and reversible factors for RA patients in whom a reconstructive procedure and joint surgery may be needed seems imperative. Few studies have addressed the topic in the T2T era and in the context of early RA patients, with conflicting results. None had been performed in Latin-American patients in whom the disease has unique characteristics. Most often, studies have focused on predictors at disease presentation and identified relevant, clinical and laboratory markers of disease activity and severity [9–12], number of copies of the shared epitope present in the patient [9, 12], radiographic damage [9, 10], demographic variables [9, 11] and short disease duration [9]. In addition, the most important time-varying factors associated with a reduced risk of joint surgery have been early treatment with conventional DMARDS during the first 2 years [10], good response to treatment during the first years of follow-up [10, 13, 14], lower annual radiographic progression rate [10, 12] and lower HAQ score at the beginning of a follow-up window [11] or in the early course of the disease [12]. Finally, the intensity of RA-specific treatment during the first year of disease diagnosis has also been associated with longer time to joint replacement surgery [13].

The objectives of the study were to describe the incidence rate of orthopedic and hand surgery indication (OHSI) in a cohort of Mexican Mestizo early RA patients treated with conventional DMARDs according to a T2T strategy (objective 1) and to investigate OHSI predictors (objective 2).

## Methods

### Setting and study population

Patients with RA were identified from the Early Arthritis Clinic (EAC) of the Instituto Nacional de Ciencias Médicas y Nutrición “Salvador Zubirán,” located in México City. When first evaluated in the clinic, patients had disease duration of less than a year and no specific rheumatic diagnosis except RA. Once enrolled, the patients were evaluated every 2 months during the first 2 years of follow-up and every 2, 4 or 6 months, thereafter. Treatment prescribed was T2T oriented; traditional DMARDs were used in 98% of the patients with/without corticosteroids (up to 55% of the patients received low doses of oral corticosteroids) during their follow-up.

At baseline evaluation, a complete medical history and demographic data were recorded along with rheumatoid factor (RF) and antibodies to cyclic citrullinated peptides (ACCP). Follow-up evaluations were standardized and included prospective assessments of swollen and tender joint counts, patient- and physician-reported outcomes [2, 3], extensive disease activity evaluation, comorbidity, treatment and persistence with therapy; complete laboratory parameters were also determined. Hand and feet X-rays were performed at baseline and thereafter every year. In addition, at follow-ups, indication of joint surgery (yes/no and date of the indication) and, when appropriate, identification of the joint(s) candidate(s) for surgery, of the intervention recommended and of the surgery date were prospectively recorded.

### Study design

Through February 2018, the cohort comprised 198 RA patients recruited from 2004 onwards; among them, 185 patients had at least fourteen months of follow-up that was required due to the case-control nested within a cohort design (the first OHSI was at 14 months of follow-up); 5 out of 185 patients (2.7%) were deceased and 43 (23.3%) were lost to follow-up while 137 (74%) had active follow-up. Charts up to the last follow-up or death, were retrospectively reviewed.

A case-control study nested within a cohort was designed to accomplish objective 2. Cases were defined as RA patients with (incidental) OHSI (see definition below). Controls (RA patients without OHSI) were paired to cases (4 controls: 1 case) according to age (± 5 years), sex, baseline RF and ACCP (absent vs. present).

### Definitions

- (Incidental) **OHSI** was corroborated by either the orthopedic surgeon or the hand surgeon, for the first time, after at least 6 months of follow-up. All of the cases had rheumatic and surgical evaluations. Surgical indication (instead of surgery) was considered due to the following reasons: Patient’s desire to delay the surgery due to costs or fears, surgical waiting list that may last up to 6-12 months, and a prosthesis donation program waiting list that may last up to 1 year. OHSI were, in all the cases, indicated for joint damage secondary to RA.

- At each follow-up evaluation, **disease activity** was graded as remission, mild, moderate and high disease activity, based on DAS28 cut-offs [15]. **Sustained remission (SR)** was defined if the patient’s DAS28-ESR was maintained at < 2.6 for at least 6 months of continuous follow-up; **time in SR** was computed from the first visit (time) that SR was achieved to the last follow-up with SR. **Flare** was arbitrarily defined as any increase in EULAR disease activity category.

- **Cumulative disease activity** was computed from baseline evaluation up to OHSI for cases or equivalent time for controls, as the mean of DAS28 at each follow-up assessment; **number of flares** was similarly computed (one patient had persistent high disease activity and the maximum number of flares/patient was arbitrarily assigned: 14 flares). Finally, **follow-up time in remission** was computed as the percentage of the entire follow-up the patient had periods of at least 6 months of follow-up with DAS28 < 2.6, either continuous or not.

- At each assessment, **persistent patients** were identified as previously published [2]. **Persistence** was defined (within each patient) as the percentage of the patient’s entire follow-up (up to OHSI for cases or equivalent for controls) that he/she was persistent with therapy.

Finally, for each patient, a precise description of cumulative DAS28, sustained remission, time in sustained remission, number flares/patient, patient category regarding being persistent (vs. not persistent) and patient’s persistence during the follow-up period was provided; treatment was also provided.

### Statistical analysis

Descriptive statistics was used. Student t test and X^2^ were used for normally distributed variables and Mann-Whitney U for non-normally distributed variables.

To achieve objective 2, baseline characteristics were first compared between OHSI patients (*N* = 12) and their counterpart (*N* = 173). In addition, a case-control study nested in the cohort was designed to compare cumulative disease activity, treatment and persistence between cases and controls. Finally, a logistic regression model was used to identify predictors of first OHSI. The selection of variables was based on their statistical significance in the bivariate analysis (*p* ≤ 0.06); variables a priori considered were disease duration (at baseline), cumulative disease activity and persistence related variables. Based on the number of outcomes of interest (*N* = 12), 3 to 4 variables were included.

All statistical tests were 2-sided and evaluated at the 0.05 significance level. Statistical analysis was performed using the SPSS/PC program (v.17.0; Chicago, IL).

### Ethics approval and consent to participate

The study was approved by the Institution’s Internal Review board “Comité de Ética del Instituto Nacional de Ciencias Médicas y Nutrición Salvador Zubirán” with reference number IRE-274-10/ 11-1. Written informed consent was obtained from all of the patients to have their charts reviewed and data presented in scientific forums or published.

## Results

### Study population characteristics (Table 1)

Patients entering the EAC were predominantly middle-aged (mean ± SD age of 38.5 ± 12.9 years) and female (87.6%), with (median, 25th–75th IQR) 5.3 (3. 3-7.0) months of symptom duration. As expected, patients had high disease activity, high disability and poor function. The majority of the patients had RF and ACCP while a few (9.7%) had erosive disease. (Median, 25th–75th IQR) Charlson score was 1 (1-1). Regarding treatment at referral to the clinic, 54.6% of the patients were indicated at least one DMARD and 38.9% low doses of oral corticosteroids (Table 1).

**Table 1:** Population characteristics at baseline and comparison between patients with/without OHSI, and between OHSI patients and their paired controls

	Population (*N* = 185)	Patients with OHSI (*N* = 12)	Patients without OHSI (*N* = 173)	OHSI-paired controls (*N* = 48)	p_1_	p_2_
Female sex, N° (%) of patients	162 (87.6)	11 (91.7)	151 (87.3)	44 (99.7)	1	1
(Mean ± SD) Age at cohort inclusion, years	38.5 ± 12.9	42 ± 15.6	38.3 ± 12.7	42 ± 14.9	0.33	1
Medium-low socioeconomic level, N° (%) of patients	165 (89.2)	11 (91.7)	154 (89)	43 (89.6)	1	1
(Mean ± SD) Years of formal education	11.2 ± 3.9	10.2 ± 4.3	11.3 ± 3.9	10.8 ± 4.2	0.32	0.66
Disease duration, months	5.3 (3. 3-7.0)	7.5 (6. 2-9.3)	5 (2. 9-7.4)	5 (2. 9-7.4)	0.02	0.02
N° (%) of patients with RF	152 (82.8)	11 (91.7)	141 (81.5)	44 (99)	0.7	1
N° (%) of patients with ACCP	158 (85.9)	12 (100)	146 (84.9)	43 (91.5)	0.22	0.6
N° (%) of patients with erosions	18 (9.7)	2 (16.7)	16 (9.2)	5 (10.4)	0.33	0.62
DAS28	5.9 (4.8-6.9)	7 (5.4-7.7)	6.3 (4.9-7.5)	6.3 (4.9-7.5)	0.27	0.27
Physician-VAS	36 (36–49)	51 (31-68)	41 (30-52)	41 (30-51.5)	0.23	0.23
CRP, mg/dL	0.7 (0.3-2.5)	3.6 (0.5-7.1)	1.2 (0.3-2.3)	1.2 (0.3-2.3)	0.06	0.06
ESR, mm/H	11 (12-39)	42 (19-74)	26 (15-49)	26 (15. 3-49)	0.14	0.14
Charlson Score	1 (1-1)	1 (1-1)	1 (1-1)	1 (1-1)	0.18	0.12
HAQ (0–3)	1.4 (0.9-2.1)	1.4 (1-2.3)	1.4 (0.9-2)	1.4 (0.9-2)	0.73	0.73
SF-36 (0–100)	38 (27-55)	33 (24-56)	34 (26-57)	33.8 (26.4-57.4)	0.94	0.94
Patient-overall disease-VAS	53 (33-76)	71 (41–92)	62 (34-78)	62 (34-78)	0.29	0.29
Pain-VAS	50 (31-73)	61 (46–98)	52 (33-78)	52 (33.3-77.8)	0.48	0.48
N° (%) of patients with DMARDs	101 (54.6)	7 (58.3)	94 (54.3)	19 (39.6)	1	1
N° (%) of patients with corticosteroids	72 (38.9)	4 (33.3)	68 (39.3)	15 (31.3)	0.77	1

*OHSI* orthopaedic and hand surgery indication, *N* number, *SD* standard deviation, *RF* rheumatoid factor, *ACCP* antibodies to cyclic citrullinated peptides, *DAS* disease activity score (28 joints evaluated), *VAS* visual analogue scale, *CRP* C reactive protein, *ESR* erythrocyte sedimentation rate, *HAQ* health assessment questionnaire, *SF-36* short form 36, *P*_*1*_ Comparison between patients with vs. without OHSI, *P*_*2*_ Comparison between patients with OHSI vs. paired controls

Data presented as median (25th–75th IQR) unless otherwise indicated

### Description of the patients with OHSI

**Fig. 1:**
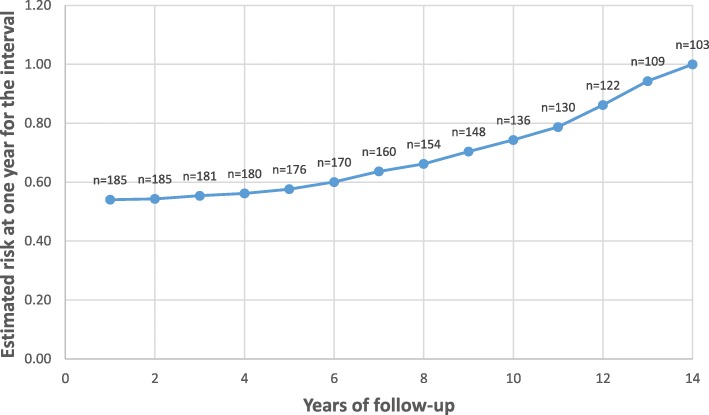
OHSI annual incident rate according to cohort follow-up. *Per 100 person years*

Through February 2018, the cohort contributed to 1538 patient-years of follow-up. There were 12 patients with incidental OHSI, 11 of them were female, their range of age was 20-66 years old and OHSI was at a follow-up of 85 ± 44.5 months; 8 patients (66.7%) received orthopedic surgery indication while 4 (33.3%) received hand surgery indication; the incident global rate was 8/1000 patient-years. Figure 1 summarizes the OHSI annual incident rate that progressively increased after the 2nd year of follow-up.

Patient description and surgery indication are summarized in Table 2, which also identifies patients already intervened, 8 patients (66.7%)**.** Half of the patients (*N* = 6) received a surgical indication between 5 and 10 years of follow-up, and 3 (25%) patients each, before 5 years of follow-up and after 10 years of follow-up.

**Table 2:** OHSI patient’s description

Number of patient	DR months	Baseline DAS28	OHSI DAS28	Follow-up to OHSI, months	Treatment at OHSI	Surgical indication
1^a^	7.3	5.6	3.4	110	MTX + SUL + LEF + PDN	Left hip arthroplasty
2^a^	4.7	7.2	2.4	114	MTX + PDN	Right knee arthroplasty
3^a^	7.6	8.7	6.4	68	MTX + PDN	Left hip arthroplasty
4^a^	1	7.1	3.0	132	MTX + PDN	Hemi-resection of the distal ulna and extensor tendon’s reconstruction (right hand)
5	8.8	7.8	2.4	146	MTX + PDN	Extensor tendon’s reconstruction (left hand)
6	6	5.8	3.4	124	CLQ + SUL + PDN	Right hip arthroplasty
7	10.5	5.3	2.5	72	MTX + CLQ + PDN	Right knee arthroplasty
8^a^	6.7	4.6	5.2	114	AZA + PDN	Extensor tendon’s reconstruction (left hand)
9^a^	9.5	8.0	2.3	66	MTX + PDN	Left shoulder arthroplasty
10^a^	7.2	7.1	2.6	52	MTX + CLQ + SUL + PDN	Right hand synovectomy
11	12.1	5.3	2.2	14	MTX + CLQ + SUL	Right knee arthroplasty
12^a^	8.5	6.8	2.5	14	MTX + CLQ + SUL + PDN	Left hip arthroplasty

*DAS28* Disease activity score (28 joints evaluated), *MTX* methotrexate, *SUL* sulphasalazine, *LEF* leflunomide, *PDN* prednisone, *CLQ* chloroquine, *AZA* azathioprine, *F* female, *M* male, *DR* months of symptom’s disease duration up to cohort baseline evaluation, *DAS28* disease activity

^a^Patients already intervened

### Comparison of baseline characteristics from patients with/without OHSI

Table 1 summarizes the results; OHSI patients had longer symptom duration and tended to have higher CRP levels than their counterparts.

### OHSI predictors (objective 2)

Data from 12 cases paired with 48 controls (1:4) are summarized in Table 1 (comparison of baseline characteristics) and Table 3 (comparison of cumulative variables). Regarding baseline characteristics, similar results as those described when OHSI were compared to their counterparts were found. Regarding cumulative variables (Table 3), OHSI patients had a significantly higher cumulative number of flares and had a lower percentage of their follow-ups in remission status. In addition, OHSI patients were less frequently persistent with therapy during follow-up and had a lower percentage of their follow-up with treatment persistence. Cumulative treatment was similar between cases and controls.

**Table 3:** Comparison of cumulative disease activity, treatment and persistence with therapy between OHSI and paired controls

	Cases (*N* = 12)	Controls (*N* = 48)	p
DAS28	3.1 (2.5-3.5)	2.6 (2.3-3.2)	0.09
N° (%) of patients who achieved a 1st SR	8 (66.7)	40 (83.3)	0.23
Months in 1st SR	12 (0–28.5)	27 (10-41.5)	0.12
N° of flares/patient	6 (3.5-9.5)	3 (1-6)	0.03
% of follow-up in remission status	50.7 (19-65.6)	71.4 (50.3-80.6)	0.02
N° DMARD/patient	1.5 (1-3)	2 (1-2.5)	0.88
N° of patients with corticosteroids	11 (91.7)	42 (87.5)	1
Dose of corticosteroids	7.5 (5-7.5)	5 (5-7.5)	0.32
N° (%) of patients persistent	2 (16.7)	24 (50)	0.05
N° (%) of patients always persistent	2 (16.7)	22 (45.8)	0.06
% of patients follow-up persistent with therapy	75 (50–82.5)	92.5 (75–100)	0.04

Data presented as median (Q25-Q75) unless otherwise indicated

*OHSI* orthopaedic and hand surgery indication, *N* number

The different multiple regression models tested included baseline variables (months of symptom disease duration) and cumulative variables (N° of flares/patient, % of follow-up in remission status, N° of patients persistent and % of follow-up patients were persistent with therapy) and yielded similar results: Longer symptom duration at referral to the EAC (OR: 1.31, 95%CI: 1.02–1.68, *p* = 0.032) and a higher number of flares (OR: 1.61, 95%CI: 1.05–1.61, *p* = 0.015) were the only predictors of OHSI.

Finally, we used ROC to define the best cut-off for symptom duration and cumulative number of flares to predict OHSI and identified 6 months (sensitivity: 0.833; specificity: 0.665; AUC: 0.746, 95% CI: 0.593–0.899) and 5 flares/patient (sensitivity: 0.750; specificity: 0.625; AUC: 0.702, 95% CI: 0.522–0.882), respectively.

### Flare distribution

**Fig. 2:**
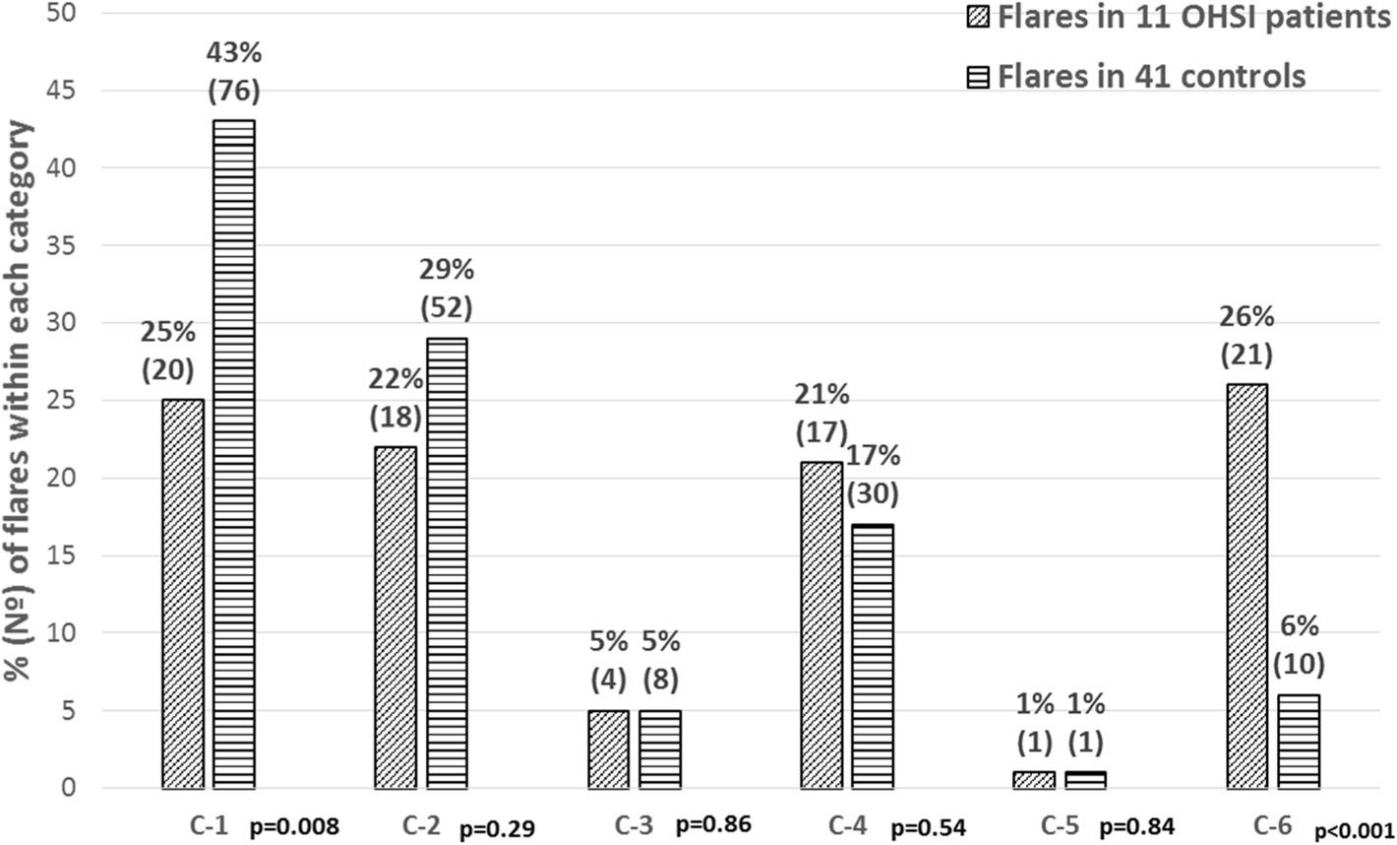
Comparison of flare category distribution between 11 OHSI patients and 41 controls. *C-1 to C-6 = category 1 to 6 flare*

Flares were divided into six categories: (1) Patients who had increased disease activity from remission status to low disease activity; (2) from remission to moderate disease activity; (3) from remission to high disease activity; (4) from low disease activity to moderate disease activity; (5) from low disease activity to high disease activity and (6) from moderate disease activity to high disease activity. Eleven patients with OHSI (one patient did not present flares during follow-up) had 81 flares while 41 controls (7 were flare-free) had 177 flares. Figure 2 summarizes the comparison of the flare-category distribution between both groups. Interestingly, the controls showed a higher number of category (1) flares compared to cases while the opposite figure was true regarding category (6) flares. At the patient level, similar tendencies were observed but differences did not reached statistical significance (data not shown).

## Discussion

The present study was developed in a dynamic cohort of Hispanic early RA patients, in whom comprehensive rheumatologic follow-up evaluations were performed from 2004 onwards. The main purpose of the study was to prospectively assess the occurrence of and predictive factors for joint surgery indication; surgical indication (instead of surgery) was chosen due to intrinsic and extrinsic sources of vulnerability from our patients, who may delay or even halt interventions because of costs [16, 17].

We found a low incident global rate of OHSI (8/1000 patient-years) after a mean follow-up of 7 years. The OHSI annual incidental rate progressively increased after the second year. In the literature, few inception cohorts of early RA had assessed rates of orthopedic and small joint surgery, with conflicting results [9–14, 18, 19]; prevalence ranged from 5.3% at a mean follow-up of 4.6 years [13] to 58% after a mean follow-up of 16 years [12]; variations may be explained by a lack of a uniform definition of “early disease” [9–14, 19], a wide spectrum of follow-ups that may last up to 25 years [19], differences in the genetic background of the populations in whom surgery was assessed (from the UK [9, 11, 14], Sweden [12, 18], Canada [13], the Netherlands [10] and Finland [19]) and the year of patient’s inclusion, which may have affected the current standard of care. Our cohort had distinctive characteristics which may additionally explain our low OHSI prevalence, estimated as 2.2% at 5 years and reaching 11.7% at last follow-up (13 years); all of our patients were of Hispanic origin and there is only one study which evaluated 355 major joint surgeries performed in Brazilian patients, although only 8 patients had RA diagnosis, which precludes any comparison [20]; also, mean symptom’s duration from our patients was close to 5 months, the cohort had a limited follow-up and included patients from 2004 onwards; finally, our patients received a T2T strategy, primarily with combined traditional DMARDs meanwhile only 4 patients had access to biologics. Of note, Moura et al. [13] identified new-onset RA patients in the Québec Health Insurance Program databases from 2002 to 2011, which is close to our cohort initiation date, and described 10.9 joint replacements during 1000 person-years, similar to our finding. Published recent-onset cohorts with a higher prevalence of joint surgery included patients one or more decades previous to our inclusion date [9–12, 14, 18, 19]. These data suggest a decline in orthopedic surgery utilization and might be explained by earlier, more aggressive and better treatment strategies [21–24]. Nonetheless, it should be mentioned that there are conflicting results regarding benefits of biological DMARDs on the need for joint replacement surgery [25, 26].

Longer symptom duration at referral and a higher number of flares/patient during follow-up were the only predictors of incidental OHSI. Early RA cohorts identified additional predictors, and the variability of the results may be explained by the cohort’s heterogeneity (as previously described), the selection of variables to be included and how the models were built. Baseline and comprehensive cumulative variables related to disease activity, to treatment and to adherence were both included in the model as a unique characteristics of our study design. Two groups of investigators, Gwinnith et al. [11] and Kapetanovic et al. [12], also assessed baseline and time varying predictors of orthopedic surgery in recent-onset RA cohorts; the former found that functional disability at time-points was the strongest predictor of future major surgery while acute reactant-phase determinations, baseline HAQ, and early radiological changes were identified as predictors of future need for large joint replacements in the second cohort. In addition, disease activity, radiographic damage, acute reactant-phase determinations, female sex and genotyping were identified as predictors of joint surgery in recent-onset RA, when only baseline variables were considered [9, 11].

In our study, the disease activity construct was extensively assessed during follow-up. Our results highlight that in order to prevent joint surgery, the number of flares must be controlled. Interestingly, we also found differences between patients with/without OHSI in the distribution of category-1 and -6 flares. The former represents a surrogate of adequate disease activity control and mild flares and was the predominant category of flares in patients without OHSI; meanwhile, category-6 flares indicate unsatisfactory disease activity control and were predominant in patients who ultimately received OHSI. Markusse et al. [27] followed up 508 RA patients from the BeSt study, who were T2T for 10 years. The authors formulated 3 definitions of disease flare based on the original DAS; patients who suffered a minor flare B showed more joint damage progression when compared with their counterpart. A higher number of flares was associated with higher disease activity [28] that, if uncontrolled, may progress to severe joint destruction, unremitting pain and joint deformity; in such clinical context, joint arthroplasty has proven to be a successful intervention to improve physical function [29–31].

In addition to flare control, symptom duration at referral was also found to prevent joint surgery and could be considered a surrogate of early use of DMARDs. Moura et al. [13] found that longer exposure to methotrexate or DMARDs during the first year of follow-up after RA diagnosis was associated with longer time to joint replacement surgery, in a new-onset cohort of 11,333 RA patients. Our patients were indicated DMARDs at first evaluation and symptom duration at referral cut-off of 6 months may be considered an early referral, which is an evidence-based recommendation in newly diagnosed RA patients who improves long-term outcomes and patient quality of life [32].

The study has limitations that must be addressed. First, the study was conducted at a single center which limits the generalization of the results. Second, it was also conducted in an observational cohort and therefore has the limitations of such cohorts, particularly follow-up losses and lack of standardization and control with respect to certain variables and outcomes [33]. Third, we arbitrarily defined and classified flares into 6 categories; it should be emphasized that at the patient level, the six categories may be present and it is unknown if a predominant category may eventually have the greatest impact; also, flare was arbitrarily defined based on a change in EULAR categories, although there is no consensus on flare’s definition, as recently published [34]; nonetheless, we performed a sensitivity analysis defining flare as DAS28 > 3.2 [34] and same patients were identified. Fourth, access to biologics was restricted and it may have impacted disease activity control. Finally, the number of OHSI patients was limited and results should be interpreted with caution.

## Conclusions

Our study complements the existing literature related to predictors of joint surgery in real world early RA patients. A delay/prevention of joint surgery may be added to the list of benefits when patients are referred early to a rheumatologist. In addition, flare attenuation should also be considered a target to impact joint interventions, particularly those flares that translate into high disease activity.

## Abbreviations

RA: Rheumatoid Arthritis
OHSI: Orthopedic and hand surgery indication
T2T: Treat-to-Target
IRB: Institutional Review Board
OR: Odds Ratio
CI: Confidence Interval
EULAR: European League Against Rheumatism
DMARDs: Disease Modifying Anti-rheumatic Drugs
HAQ: Health Assessment Questionnaire
EAC: Early Arthritis Clinic
RF: Rheumatoid Factor
ACCP: Antibodies to Cyclic Citrullinated Peptides
DAS28: Disease Activity Score on 28 Joints
ESR: Erythrocyte Sedimentation Rate
SR: Sustained Remission
SPSS/PC: Statistical Package for the Social Sciences
CRP: C Reactive Protein
ROC: Receiver Operating Characteristic
AUC: Area Under Curve
